# A mathematical model to estimate the state-specific impact of the Health Resources and Services Administration’s Ryan White HIV/AIDS Program

**DOI:** 10.1371/journal.pone.0234652

**Published:** 2020-06-22

**Authors:** Pamela W. Klein, Stacy M. Cohen, Evin Uzun Jacobson, Zihao Li, Glenn Clark, Miranda Fanning, Rene Sterling, Steven R. Young, Stephanie Sansom, Heather Hauck

**Affiliations:** 1 HIV/AIDS Bureau, Health Resources and Services Administration, Rockville, Maryland, United States of America; 2 Division of HIV/AIDS Prevention, Centers for Disease Control and Prevention, Atlanta, Georgia, United States of America; Brigham and Women’s Hospital, UNITED STATES

## Abstract

**Background:**

Access to and engagement in high-quality HIV medical care and treatment is essential for ending the HIV epidemic. The Health Resources and Services Administration’s (HRSA) Ryan White HIV/AIDS Program (RWHAP) plays a critical role in ensuring that people living with diagnosed HIV (PLWH) are linked to and consistently engaged in high quality care and receive HIV medication in a timely manner. State variation in HIV prevalence, the proportion of PLWH served by the RWHAP, and local health care environments could influence the state-specific impact of the RWHAP. This analysis sought to measure the state-specific impact of the RWHAP on the HIV service delivery system and health outcomes for PLWH, and presents template language to communicate this impact for state planning and stakeholder engagement.

**Methods and findings:**

The HRSA’s HIV/AIDS Bureau (HAB) and the Centers for Disease Control and Prevention‘s Division of HIV/AIDS Prevention (CDC DHAP) have developed a mathematical model to estimate the state-specific impact of the RWHAP. This model was parameterized using RWHAP data, HIV surveillance data, an existing CDC model of HIV transmission and disease progression, and parameters from the literature. In this study, the model was used to analyze the hypothetical scenario of an absence of the RWHAP and to calculate the projected impact of this scenario on RWHAP clients, RWHAP-funded providers, mortality, new HIV cases, and costs compared with the current state inclusive of the RWHAP. To demonstrate the results of the model, we selected two states, representing high HIV prevalence and low HIV prevalence areas. These states serve to demonstrate the functionality of the model and how state-specific results can be translated into a state-specific impact statement using template language.

**Conclusions:**

In the example states presented, the RWHAP provides HIV care, treatment, and support services to a large proportion of PLWH in each state. The absence of the RWHAP in these states could result in substantially more deaths and HIV cases than currently observed, resulting in considerable lifetime HIV care and treatment costs associated with additional HIV cases. State-specific impact statements may be valuable in the development of state-level HIV prevention and care plans or for communications with planning bodies, state health department leadership, and other stakeholders. State-specific impact statements will be available to RWHAP Part B recipients upon request from HRSA’s HIV/AIDS Bureau.

## Introduction

Recent biomedical advances have established HIV as a chronic, manageable condition, and people living with diagnosed HIV (PLWH) can have a near normal life expectancy [1]. Regular access to HIV medical care, treatment, and support services, in addition to consistent adherence to HIV treatment regimens, greatly reduces HIV-associated morbidity and mortality [2, 3]. Moreover, PLWH who take HIV medication as prescribed and achieve and maintain a suppressed viral load have effectively no risk of sexually transmitting HIV to their HIV-negative partners [4]. For these reasons, access to and engagement in high quality and comprehensive HIV medical care and treatment are essential for ending the HIV epidemic [5].

In the United States, the Ryan White HIV/AIDS Program (RWHAP), administered by the Health Resources and Services Administration (HRSA) and funded for nearly $2.4 billion in FY 2020, provides a comprehensive system of HIV care, treatment, and support services to more than half of PLWH living in the U.S. and their affected family members each year [6]. Through funding awarded to cities, states, and community-based organizations for the delivery of core medical and support services, HRSA’s RWHAP plays a critical role in ensuring that PLWH are linked to and consistently engaged in high-quality care and receive HIV medication in a timely manner. Low-income PLWH receiving HIV health care through RWHAP have higher rates of HIV viral suppression compared to PLWH who do not receive RWHAP services [7]. Therefore, the HIV medical services and treatments supported by RWHAP have the potential to prevent mortality and HIV transmissions and contribute to efforts to end the HIV epidemic in the U.S.

A key feature of the RWHAP is its ability to respond to the unique HIV-related needs of many populations of PLWH across diverse settings in the U.S., including all 50 states, the District of Columbia, Puerto Rico, the U.S. Virgin Islands, and six U.S. Pacific Territories/Associated Jurisdictions. HRSA RWHAP grants, awarded to metropolitan areas, states/territories, and local clinics and community-based organizations, allow for RWHAP funding to be responsive to the needs and distinct features of local HIV epidemics. However, state-level variation in HIV prevalence, the proportion of PLWH served by the RWHAP, and local health care infrastructure could influence the impact of the RWHAP within a state.

The HRSA HIV/AIDS Bureau (HAB) and the Centers for Disease Control and Prevention Division of HIV/AIDS Prevention (CDC DHAP) developed a mathematical model to estimate the state-specific impact of the RWHAP. The model was used to examine the hypothetical scenario of an absence of the RWHAP and calculated the impact of this scenario on RWHAP clients, RWHAP-funded providers, and projected mortality, transmissions, and costs compared with the current state of the RWHAP. This paper describes the components of the model and presents results from two example states, one with high HIV prevalence and one with low HIV prevalence. The results for these example states are presented in a state-specific impact statement template developed as a resource for states to demonstrate the impact of the RWHAP within their states for planning and stakeholder engagement.

## Methods

### Ryan White HIV/AIDS program and data sources

The RWHAP has five statutorily defined Parts that provide funding for medical and support services, technical assistance, clinical training, and the development of innovative models of care to meet the needs of different communities and populations affected by HIV. Part A provides funding to Eligible Metropolitan Areas and Transitional Grant Areas that are most severely affected by the HIV epidemic. Part B provides funding to all 50 states, the District of Columbia, Puerto Rico, the U.S. Virgin Islands, and six U. S. Pacific Jurisdictions. Part B also includes funding awards for the AIDS Drug Assistance Program (ADAP) to support medication and insurance assistance. Part C provides funding to local community-based organizations, community health centers, health departments, academic medical centers, and hospitals in the U.S., while Part D provides funding to support services for low-income women, infants, children, and youth living with HIV and their affected family members. Part F includes the Special Projects of National Significance, AIDS Education and Training Centers, and Dental Programs; Part F programs are not included in this model [6].

RWHAP data used for this model were from the Ryan White HIV/AIDS Services Report (RSR) and the ADAP Data Report (ADR). The RSR dataset is HRSA HAB’s primary source of annual, client-level RWHAP data from Parts A-D recipients, excluding ADAP data, and is used to assess the numbers and demographics of clients receiving services, as well as their HIV-related outcomes. The ADR provides similar client-level information about individuals served and services delivered through ADAPs. Each year, grant recipients and subrecipients that receive RWHAP funds to provide core medical services, support services, or ADAP services are required to submit data to HRSA. Data collection through RSR and other RWHAP data sources is a routine program activity and the data are used for program monitoring, improvement, evaluation, and policy purposes only. Therefore it is not human subject research and does not require IRB review and approval.

### Model overview

The state-specific impact model consists of four components:

1. Current number and proportion of PLWH reached by RWHAP
2. Estimated number of clients and provider organizations impacted by the absence of the RWHAP
3. Projected number of additional deaths attributable to the absence of the RWHAP
4. Projected number of additional new cases and associated HIV care and treatment costs attributable to the absence of the RWHAP

This model provides an estimate of the state-specific impact of the RWHAP under the current state of the HIV epidemic and the RWHAP. The “current state” is defined as the most recent year for which RWHAP and CDC surveillance data are available at the time of running the model for each specific state; the specific years of data used in this demonstration are provided in Table 1. Therefore, the model does not take into account any potential changes to the RWHAP’s legislative authorization or funding levels. Additionally, the model does not incorporate potential scientific, policy, or programmatic advancements that may impact the trajectory of HIV care in the future. The model uses a relatively short time horizon (1-year and 5-years) to mitigate the impact of these unknown future developments on model projections. The model was created using Microsoft Excel.

**Table 1:** Model parameters, underlying data elements, and data sources.

Parameter	Data Element	Source(s)
***Component 1—Current reach of RWHAP***		
**No. of RWHAP clients**	RWHAP Parts A-D clients in each state	2017 RSR [6]
	RWHAP ADAP clients in each state	2017 ADR
	Estimated RSR/ADR overlap in each state	State-provided, or nationally-averaged probabilistic value
**% of PLWH served by the RWHAP**	No. people aged 13 years and older living with diagnosed HIV in each state	NCHHSTP AtlasPlus [9]
***Component 2—Clients & Providers Impacted by the hypothetical absence of the RWHAP***		
**No. of RWHAP clients impacted**	Uninsured RWHAP Parts A-D clients in each state	2017 RSR [6]
	RWHAP clients receiving insurance premium assistance in each state	2017 ADR
**No. of RWHAP-funded providers impacted**	RWHAP-funded provider organizations in each state	State-provided, or state-specific 2017 RSR value
***Component 3—Projected number of additional deaths attributable to the hypothetical absence of the RWHAP***		
**No. of additional deaths**	Care-continuum model framework calculated for each state	Gopalappa 2017 [12]
	National-level all-cause mortality rates (annual probability) Not in care: 2.48% Receiving HIV care but not virally suppressed: 1.61% Virally suppressed: 1.39%	Khurana 2018 [11]
	No. of RWHAP clients at each care-continuum step in each state	2017 RSR [6]
**Average No. of deaths**	No. of deaths occurring per year in each state	NCHHSTP AtlasPlus [9]
***Component 4—Projected number of additional cases and associated HIV care and treatment costs attributable to the hypothetical absence of the RWHAP***		
**No. of additional HIV cases**	Care-continuum model framework calculated for each state	Gopalappa 2017 [12]
	National-level transmission rates (per 100 person-years) Not in care: 6.6, 95% CI: 6.5–6.7 Receiving HIV care but not virally suppressed: 6.1, 95% CI: 6.0–6.3 Virally suppressed: 0.0, 95% CI: 0.0–0.0	Li 2019 [13]
	No. of RWHAP clients at each care-continuum step in each state	2017 RSR [6]
**Average No. of HIV cases**	No. of HIV cases diagnosed per year in each state	NCHHSTP AtlasPlus [9]
**Additional costs**	National-level lifetime HIV care and treatment costs per client $477,673, 95% CI: $474,506-$480,839 ($US 2017)	Farnham 2013 [14]

We describe the methodology for each component separately here. All parameter inputs are detailed in Table 1 and formulas are provided in S1 Appendix.

#### Component 1: Current reach of the RWHAP

The model first calculates the total number of PLWH served by the RWHAP (“RWHAP clients”) and the percentage of PLWH within the state served by the RWHAP during the specified year.

The total number of RWHAP clients includes those who received either RWHAP services, ADAP services, or a combination of the two, during the most recent calendar year. This value was calculated as the sum of clients in the RSR (which includes non-ADAP data from RWHAP Parts A-D) and the ADR datasets minus the estimated overlap between the datasets [6]. For this analysis, the estimated overlap between the datasets was derived using the results of a national-level conditional probability model to estimate the proportion of clients in the ADR dataset who are also present in the RSR dataset [8]. “State-level” refers to all clients receiving services from RWHAP-funded entities in that state, regardless of the Part funding through which they were served. That is, the model values are agnostic to funding streams, represent more than the number of clients served using Part B or state-administered funding, and reflect the state in which a client received services rather than their state of residence.

The percentage of PLWH within the state served by the RWHAP was calculated as the total number of RWHAP clients divided by the number of people aged 13 years and older living with diagnosed HIV infection in the state, as reported by the most recently available data from the CDC’s National HIV Surveillance System (NHSS) [9]. These measures do not align precisely; RWHAP clients counted for the state in which they received services, while surveillance data categorizes PLWH based on their state of residence. Although the RWHAP serves clients younger than 13 years of age, publicly available NHSS state-level data do not include PLWH younger than 13 years old. PLWH younger than 13 years represent 4.2% of the U.S. HIV epidemic and 0.9% of RWHAP clients; therefore, the inclusion of PLWH under age 13 in the numerator but their exclusion in the calculation denominator is unlikely to significantly bias the calculations [10].

#### Component 2: Estimated number of clients and providers impacted by the absence of the RWHAP

To calculate the number and percentage of RWHAP clients in the state who would be impacted by the absence of the RWHAP, we assumed that, at a minimum, uninsured RWHAP Part A-D clients and ADAP clients receiving insurance premium assistance would lose access to HIV-related medical care and treatment. We assumed that uninsured ADAP clients would comprise the majority of the overlap between the RSR and ADR datasets. Therefore, to avoid double counting the large proportion of clients who access both RWHAP Part A-D and ADAP services, uninsured ADAP clients were not added to the estimated number of clients impacted by the absence of the RWHAP. This estimated number and percentage of RWHAP clients impacted by the hypothetical absence of the RWHAP assumes that these PLWH would have no alternate mechanism to access HIV care and treatment without the RWHAP. The model also assumes no changes from the current health care system; that is, the model assumes that there the health care coverage options within a state do not change in response to the hypothetical absence of the RWHAP in order to provide HIV medical care and treatment to these impacted PLWH. The availability of other health care coverage options within the state would be the driver of differential values of this parameter input.

We also assumed that the absence of the RWHAP within the state would impact all providers supported by the RWHAP. In the RWHAP, a “provider” is an organization delivering RWHAP-funded services, not an individual person. As a starting point, we used RSR to identify the number of HIV providers in the state who receive RWHAP funding. HRSA HAB will work closely with states to identify whether additional organizations should be included so that the model most accurately reflects the state’s system of HIV care.

#### Component 3: Projected number of additional deaths attributable to the absence of the RWHAP

The impact of an absence of the RWHAP on mortality among PLWH was estimated using parameters from the CDC’s HIV Optimization and Prevention Economics (HOPE) model, which simulates the sexually active US population aged 13 to 64 years [11]. These mortality rates based on unpublished data from the North American AIDS Cohort Collaboration on Research and Design (NA-ACCORD) and were calibrated to CDC surveillance data, as described in the appendix of Khurana, et al.

For this analysis, the relevant stages of the HIV care continuum were “not receiving care”, “receiving care but not virally suppressed”, and “virally suppressed.” RWHAP clients were classified as “receiving care” if they received at least one outpatient ambulatory health service (OAHS) visit in the most recent calendar year, and further classified as “receiving care but not virally suppressed” or “virally suppressed” based on RWHAP state-specific proportions of clients achieving viral suppression.

We used the percentage of RWHAP clients impacted by an absence of the RWHAP calculated in Component 2 as the extent to which the current RWHAP HIV care continuum was redistributed. To obtain the redistributed HIV care continuum, the number of RWHAP clients in the “receiving care but not virally suppressed” and “virally suppressed” care-continuum stages was multiplied by the complement of the percentage of RWHAP clients impacted by an absence of the RWHAP. The number of clients by which the “receiving care but not virally suppressed” and “virally suppressed” care-continuum stages was decreased were reclassified as “not receiving care.”

To calculate the number of deaths under the current RWHAP scenario and the scenario associated with an assumed reduction in PLWH receiving HIV care, the number of people in each care-continuum stage was multiplied by the HIV care-continuum stage-specific mortality rates from Khurana, et al [11]. Additional deaths among PLWH attributable to the absence of the RWHAP over one year were calculated as the difference between the estimated number of deaths in each scenario. The number of additional deaths attributable to a reduction in PLWH receiving care over 5 years was calculated by multiplying the 1-year estimate by 5.

The 1-year average number of deaths among PLWH was calculated as the average number of deaths from the 5 most recent years of data, as reported by CDC NHSS [9]. The 5-year average number of deaths was calculated as the total number of deaths from the 5 most recent years of data, as reported by CDC NHSS. States may also request that the number of additional deaths attributable to a reduction in PLWH receive care over either time frame be presented as a percentage increase or decrease from historical mortality data.

#### Component 4: Projected number of additional cases and associated HIV care and treatment costs attributable to the absence of the RWHAP

The impact of an absence of the RWHAP on HIV transmission was estimated using parameters from the Progression and Transmission of HIV/AIDS (PATH 2.0) model developed by CDC [12, 13]. The PATH 2.0 model is an agent-based model that simulates new HIV transmissions to calculate transmission rates. These transmission rates correspond to stages of the HIV care continuum; for this analysis, the relevant stages of the HIV care continuum were “not receiving care”, “receiving care but not virally suppressed”, and “virally suppressed.”

As in Component 3, RWHAP clients were classified as “receiving care” if they received at least one OAHS visit in the most recent calendar year, and further classified as “receiving care but not virally suppressed” or “virally suppressed” based on state-specific proportions of clients achieving viral suppression. The number of RWHAP clients in the “receiving care but not virally suppressed” and “virally suppressed” care-continuum stages was multiplied by the complement of the percentage of RWHAP clients impacted by an absence of the RWHAP; those clients impacted by the absence of the RWHAP were reclassified as “not receiving care.”

To calculate the number of transmissions under the current RWHAP scenario and the scenario associated with an assumed reduction in PLWH receiving HIV care, the number of people in each care-continuum stage was multiplied by the HIV care-continuum stage-specific transmission rates from Li, et al [13]. Additional new HIV transmissions attributable to the absence of the RWHAP over one year were calculated as the difference between the estimated number of HIV transmissions in each scenario. The number of additional new HIV transmissions attributable to a reduction in PLWH receiving care over 5 years was calculated by multiplying the 1-year estimate by 5.

The 1-year average number of new HIV cases was calculated as the average number of new diagnoses of HIV infection from the 5 most recent years of data, as reported by CDC NHSS [9]. The 5-year average number of new HIV cases was calculated as the total number of new diagnoses of HIV infection from the 5 most recent years of data, as reported by CDC NHSS. States may also request that the number of addition HIV cases attributable to a reduction in PLWH receive care over either time frame be presented as a percentage increase or decrease from historical diagnosis data.

To calculate the additional lifetime HIV care and treatment costs associated with additional HIV cases, the lifetime cost of HIV care and treatment per PLWH was multiplied by the number of additional new HIV transmissions attributable to a removal of support for the RWHAP. The lifetime cost of HIV care and treatment per PLWH was derived from CDC cost estimates for PLWH in the U.S. for a person with high CD4 count at HIV diagnosis (501–900 copies/mL) [14]. These are discounted lifetime cost estimates that reflect the provider perspective and have been updated to $USD 2017. These costs are inclusive of costs for ART, medications for conditions not directly related to HIV, opportunistic infection prophylaxis, quarterly CD4 and viral load testing, HIV genotype testing at initiation of first ART regimen, and inpatient, outpatient, and emergency department utilization.

### High and low HIV prevalence states

To demonstrate the results of the model, we selected two states, which represent a high HIV prevalence (“State ‘High’”) and a low HIV prevalence state (“State ‘Low’”). We applied the calculations of each component to the state-specific data from each source. However, in order to ensure anonymity for the states, we have masked the data for each by varying all publicly available state-specific parameter inputs by up to 10% above or below their true value.

The model can present both 1-year and 5-year projections for Components 3 and 4, based on state preference. For demonstration purposes, the impact statements presented in the results section include only the 1-year projections for the high- and low-prevalence states. The quantitative 1-year and 5-year projections for both states can be found in Table 2. Similarly, for demonstration purposes, the comparison of deaths and new HIV cases to HIV surveillance data are presented as counts rather than percentages.

**Table 2:** Results of state-specific impact model in two example states (high and low prevalence).

**Component 1: Current reach of RWHAP**				
	*High Prevalence State*		*Low Prevalence State*	
No. of RWHAP clients	13,284		2,053	
% of PLWH	65.9%		83.3%	
**Component 2: Clients & Providers Impacted by the hypothetical absence of the RWHAP**				
	*High Prevalence State*		*Low Prevalence State*	
No. of RWHAP clients impacted	4,764		615	
% of RWHAP clients impacted	35.9%		30.0%	
No. of Providers Impacted	30		27	
**Component 3: Projected number of additional deaths attributable to the hypothetical absence of the RWHAP**				
	*High Prevalence State*		*Low Prevalence State*	
	1 year	5 year	1 year	5 year
No. of additional deaths	30	147	4	22
Average # of deaths	416	2,368	43	222
**Component 4: Projected number of additional cases and associated HIV care and treatment costs attributable to the hypothetical absence of the RWHAP**				
	*High Prevalence State*		*Low Prevalence State*	
	1 year	5 year	1 year	5 year
No. of additional HIV cases	172 (169–175)	860 (848–873)	27 (26–27)	133 (131–135)
Average No. of HIV cases	1,132	5,354	126	615
Additional lifetime HIV care and treatment costs	$82,159,756	$410,798,780	$12,897,171	$63,530,509
	($81,615,032-$82,704,308)	($408,075,160-$413,521,540)	($12,811,662-$12,982,653)	($62,109,298-$63,951,587)

## Results

Based on the calculations described above, the following impact statements were developed for the high HIV prevalence state and low HIV prevalence state. Although many acronyms contained within the template language were defined earlier, they are included in this section in full because the impact statements are designed to be read outside the context of this manuscript.

### Component 1: Current reach of the RWHAP

#### High HIV prevalence state

In 2017, the Health Resources and Services Administration’s Ryan White HIV/AIDS Program (RWHAP), including the AIDS Drug Assistance Program, supported direct health care, support services, and/or medication access *for 13*,*284 people living with diagnosed HIV (PLWH) in State “High”*, *representing 65*.*9% of PLWH in State “High”*.

#### Low HIV prevalence state

In 2017, the Health Resources and Services Administration’s Ryan White HIV/AIDS Program (RWHAP) which includes the AIDS Drug Assistance Program, supported direct health care, support services, and/or medication access *for 2*,*053 people living with diagnosed HIV (PLWH) in State “Low”*, *representing 83*.*3% of PLWH in State “Low”*.

### Component 2: Clients and providers impacted by an absence of the RWHAP

#### High HIV prevalence state

In the absence of the RWHAP for State High, at least *4*,*764 RWHAP clients (35*.*9% of RWHAP clients)* and *all 30 HIV providers* supported by the RWHAP would be negatively impacted. This absence of support would greatly reduce the number of PLWH who are receiving HIV medical services and treatment in State “High”.

#### Low HIV prevalence state

In the absence of the RWHAP for State Low, at least *615 RWHAP clients (30*.*0% of RWHAP clients)* and *all 27 HIV providers* supported by the RWHAP would be negatively impacted. This absence of support would greatly reduce the number of PLWH who are receiving HIV medical services and treatment in State “Low”.

### Component 3: Projected number of additional deaths attributable to the absence of the RWHAP

#### High HIV prevalence state

For PLWH, receipt of HIV treatment improves quality of life, increases life expectancy, and reduces morbidity and mortality. The reduction in PLWH receiving HIV medical services and treatment could result in *30 additional deaths* among PLWH in State “High” *over 1 year*, *above and beyond the average number of deaths otherwise expected during a 1-year period (416)*.

#### Low HIV prevalence state

For PLWH, receipt of HIV treatment improves quality of life, increases life expectancy, and reduces morbidity and mortality. The reduction in PLWH receiving HIV medical services and treatment could result in *4 additional deaths* among PLWH in State “Low” *over 1 year*, *above and beyond the average number of deaths otherwise expected during a 1-year period (approximately 43)*.

### Component 4: Projected number of additional cases and associated HIV care and treatment costs attributable to the absence of the RWHAP

#### High HIV prevalence state

Receipt of HIV treatment also prevents HIV transmission. The reduction in PLWH receiving HIV medical services and treatment could result in *172 additional HIV cases* in State “High” *over 1 year*, *above and beyond the average number of new HIV cases otherwise expected during a 1-year period (approximately 1*,*132)*. These 172 additional HIV cases could result in approximately *$82*,*160*,*000 additional lifetime HIV care and treatment costs*.

#### Low HIV prevalence state

Receipt of HIV treatment also prevents HIV transmission. The reduction in PLWH receiving HIV medical services and treatment could result in *27 additional HIV cases* in State “Low” *over 1 year*, *above and beyond the average number of new HIV cases otherwise expected during a 1-year period (approximately 126*). These 27 additional HIV cases could result in approximately *$12*,*900*,*000 additional lifetime HIV care and treatment costs*.

## Discussion

In this paper, we applied a mathematical model to estimate the state-specific impact of the RWHAP. In the example states presented, the RWHAP provides HIV care, treatment, and support services to a large proportion of PLWH. The absence of the RWHAP in these states would, at a minimum, negatively impact approximately one-third of PLWH in each of the states. The absence of the RWHAP could result in substantially more deaths and HIV cases than currently observed, resulting in considerable lifetime HIV care and treatment costs associated with additional HIV cases. The selected example states highlight how the magnitude of the model’s results can vary by HIV prevalence, the proportion of PLWH served by the RWHAP, the number of RWHAP-funded providers, and the state’s HIV care continuum.

HRSA HAB, in partnership with CDC DHAP, created this state-specific impact model as an optional resource for RWHAP Part B grant recipients to demonstrate the impact of the RWHAP within their state. RWHAP Part B grant recipients are encouraged to use contextual information from multiple sources for programmatic decision-making and planning, including HIV Surveillance, RWHAP RSR and ADR data, and other data sources or tools. This model may complement existing resources to support states in meaningful data utilization. The state-specific impact statements may be valuable in the development of state-developed “Integrated HIV Prevention and Care Plans including the Statewide Coordinated Statements of Need”, which outline state plans to facilitate the coordination, integration, and effective linkages of resources across HIV prevention and care [15]. Additionally, states may wish to use the impact statements for programmatic or policy communications with planning bodies, state health department leadership, and other stakeholders. Although the term “state” is used throughout this paper, the model can also be applied to the District of Columbia, Puerto Rico, the U.S. Virgin Islands, and Guam, all of which are funded by RWHAP Part B and submit client-level RSR and ADR data.

State-specific impact statements and model results will be available to RWHAP Part B recipients upon request, who in turn may share with other RWHAP recipients, subrecipients, and external stakeholders at their discretion. Collaboration with each state will be crucial to ensure the validity of the state-specific results. In particular, state input will be necessary for accurate information on the number of providers within each state, the estimated overlap between RWHAP clients reported in the RSR and ADR, and validation of other state-specific data elements. Additionally, HRSA HAB will provide technical support to states in the interpretation of the model results, and follow-up with states to discuss the utility of results and improvements for future work.

Updates to the model and accompanying statement will incorporate most recent HRSA and CDC data and PATH model features, including methodology and parameters. This version of the model includes sensitivity analyses for some key parameters; the model will be revised to accommodate additional sensitivity analyses as data become available. In addition, future versions of the model will adjust projections to account for the potential underestimation of RWHAP clients, including RWHAP clients who do not access medical care through the RWHAP and who may be in care elsewhere (i.e., RWHAP clients who only receive non-medical RWHAP services and may receive medical services by non-RWHAP providers), or those not reported to the RWHAP due to changes in reporting requirements. Depending on the state, the inclusion of these individuals could result in a greater estimated impact of the RWHAP. HRSA HAB is currently assessing care utilization among this population through an in-progress, multi-year study.

The focus of the model on HIV medical care and treatment may not fully account for the impact of non-medical support services funded through the RWHAP. Support services, such as case management, housing, mental health services, substance use treatment, and medical transportation, may play a significant role in retaining and engaging PLWH in HIV care. Both medical and support services likely have an impact on quality of life; however, given the lack of primary data demonstrating the specific impact of RWHAP services on quality of life, quality of life is not included in this model. Another mathematical model under development will quantify the cost-effectiveness of the entire RWHAP comprehensive system of care, including the impact of the RWHAP on quality-adjusted life years.

As with any mathematical model, assumptions were made to facilitate calculations, as detailed in the Methods section. The primary assumption is that at a minimum, uninsured RWHAP Part A-D clients and ADAP clients receiving insurance premium assistance would lose access to HIV-related medical care and treatment in the absence of the RWHAP; this may be an underestimate of the true number of RWHAP clients impacted by the absence of the RWHAP. The result of this assumption is the driver of the projection calculations in model Components 3 and 4 and yields conservative estimates. Nationally, only 20.2% of RWHAP Part A-D clients are uninsured; the remainder access RWHAP services to address gaps in their existing health care coverage and would also be impacted by the absence of the RWHAP. Therefore, the number of RWHAP clients potentially impacted, and additional deaths and cases is likely an underestimate of what would actually occur in the absence of the RWHAP.

This model indicates the potential impact of the absence of the RWHAP on RWHAP-funded provider organizations, but does not go into great depth on the implications of this impact. In the absence of the RWHAP, provider organizations may be forced to reduce the volume of services they provide to PLWH, leaving key medical and support service needs unmet. Other provider organizations might decrease the size of their workforce, which could result in a loss of experienced HIV medical providers and their institutional knowledge. Provider organizations often contribute to the growth and development of communities for PLWH and provide important employment opportunities for PLWH. The focus of this model on client-related outcomes is not meant to diminish the importance of RWHAP provider organizations, but rather reflects the lack of data to quantify the impact of the RWHAP.

Additionally, this model provides an estimate of the state-specific impact of the RWHAP under the current state of the HIV epidemic and the current reach of the RWHAP’s comprehensive system of care. As such, the model does not project the potential impact of a future increase in RWHAP funding or other initiatives to increase HIV diagnosis, linkage to and engagement in care, and viral suppression among PLWH. State-led and national initiatives to end the HIV epidemic may have impact on the HIV care landscape within a jurisdiction beyond that which is included in the current model. By using the most recently available RWHAP data and HIV surveillance data and collaborating closely with states, HRSA HAB will be able to update the model projections to account for underlying changes in the U.S. HIV epidemic, such as decreases in HIV transmission due to increases in pre-exposure prophylaxis (PrEP) prescription or increases in sustained viral suppression. Additionally, HRSA HAB will consider incorporating systems-level changes in the RWHAP or the health care infrastructure into future versions of the model, as well as working with states to customize the impact statement to account for state-specific contextual factors.

This model and its accompanying template language can serve as a resource and tool for states. HRSA HAB is committed to providing RWHAP Part B grant recipients with information to help drive decision-making and resources to assist with the implementation of HIV care and treatment programs. In addition to this state-specific impact model and template language, HRSA HAB is developing interactive data dashboards to allow all RWHAP recipients to use their own data, compare their data to state and national benchmarks, and communicate their data to stakeholders. HRSA HAB also recently partnered with the CDC and Centers for Medicare and Medicaid Services to form the HIV Health Improvement Affinity Group, a state-level collaborative effort to improve HIV health outcomes among Medicaid beneficiaries, which resulted in a publicly available toolkit [16, 17].

Partnerships among federal agencies and with state grant recipients are crucial to the development of resources and tools to address the HIV epidemic. This model and template statement serve as one example of how these partnerships can assist states in demonstrating the impact of the RWHAP system of care within their jurisdictions. By quantifying the impact of the RWHAP and communicating that impact to key stakeholders within their communities, states can demonstrate the powerful benefits of treatment as prevention and further support efforts to end the HIV epidemic.

## Supporting information

S1 Appendix State-specific impact model formulas. (DOCX)
