# Supplementary material for: A mathematical model to estimate the state-specific impact of the Health Resources and Services Administration’s Ryan White HIV/AIDS Program
Source: PLoS One. 2020 Jun 22;15(6):e0234652. doi: 10.1371/journal.pone.0234652 (PMC7307736; doi:10.1371/journal.pone.0234652)
Supplement: S1 Appendix — (DOCX) [file pone.0234652.s001.docx]

**Appendix S1: State-specific impact model formulas**

**Notation**

$$j=jurisdiction or state$$

$$s=HIV care continuum stage \left\{ \begin{aligned} not receiving care \\ receiving care but not virally suppressed \\ virally suppressed \end{aligned} \right.$$

**Component 1: Current reach of the RWHAP**

$$\boldsymbol{Number of RWHAP clients}_{\boldsymbol{j}}={Number of RWHAP nonADAP clients}_{j}+{Number of ADAP clients}_{j}-{Estimated overlap between RWHAP nonADAP and ADAP clients}_{j}$$

$$\boldsymbol{Percent of PLWH served by RWHAP}_{\boldsymbol{j}}={Number of RWHAP clients}_{j}\div{Number of people living with diagnosed HIV}_{j}$$

**Component 2: Estimated number of clients and providers impacted by the absence of the RWHAP**

$$\boldsymbol{Number of RWHAP clients impacted}_{\boldsymbol{j}}={Number of uninsured RWHAP nonADAP clients}_{j}+{Number of ADAP clients receiving insurance premium assistance}_{j}$$

$$\boldsymbol{Percent of RWHAP clients impacted}_{\boldsymbol{j}}={Number of RWHAP clients impacted}_{j}\div{Number of RWHAP clients}_{j}$$

**Component 3: Projected number of additional deaths attributable to the absence of the RWHAP**

$$\boldsymbol{Number of additional deaths (1 year)}_{\boldsymbol{j}}=\sum_{s} \left( {Change in number of clients}_{s,j} \right)\times\left( {Mortality rate}_{s} \right)$$

If $s=HIV care continuum stage \left\{ \begin{aligned} receiving care but not virally suppressed \\ virally suppressed \end{aligned} \right.$

$${Change in number of clients}_{s,j}={Reduction in number of clients}_{s,j}=-\left( {\% clients impacted}_{j} \right)\times\left( {Current number of clients}_{s,j} \right)$$

If $s=HIV care continuum stage \left\{ \begin{aligned} not in care \end{aligned} \right.$

$${{Change in number of clients}_{s,j}=Increase in number of clients}_{s,j}=\left( {Reduction in number of virally suppressed clients}_{j} \right)+\left( {Reduction in number of clients receiving care but not virally suppressed}_{j} \right)$$

$$\boldsymbol{Number of additional deaths (5 years)}_{\boldsymbol{j}}=5\times{Total number of additional deaths (1 year)}_{j}$$

$$\boldsymbol{Average number of deaths among PLWH (1 year)}_{\boldsymbol{j}}=average\left( {Number of deaths among PLWH in 5 most recent years}_{j} \right)$$

$$\boldsymbol{Total number of deaths among PLWH (5 years)}_{\boldsymbol{j}}=\sum{Number of deaths among PLWH in the 5 most recent years}_{j}$$

**Component 4: Projected number of additional cases and associated HIV care and treatment costs attributable to the absence of the RWHAP**

$$\boldsymbol{Number of additional cases (1 year)}_{\boldsymbol{j}}=\sum_{s} \left( {Difference in number of clients}_{s,j} \right)\times\left( {Transmission rate}_{s} \right)$$

If $s=HIV care continuum stage \left\{ \begin{aligned} receiving care but not virally suppressed \\ virally suppressed \end{aligned} \right.$

$${Difference in number of clients}_{s,j}=-\left( {\% clients impacted}_{j} \right)\times\left( {Current number of clients}_{s,j} \right)$$

If $s=HIV care continuum stage \left\{ \begin{aligned} not in care \end{aligned} \right.$

$${Difference in number of clients}_{s,j}=\left( {Difference in number of virally suppressed clients}_{j} \right)+\left( {Difference in number of clients receiving care but not virally suppressed}_{j} \right)$$

$$\boldsymbol{Number of additional cases (5 years)}_{\boldsymbol{j}}=5\times{Total number of additional cases (1 year)}_{j}$$

$$\boldsymbol{Average number of new HIV cases among PLWH (1 year)}_{\boldsymbol{j}}=average\left( {Number of new diagnoses among PLWH in 5 most recent years}_{j} \right)$$

$$\boldsymbol{Total number of new HIV cases among PLWH (5 years)}_{\boldsymbol{j}}=\sum{Number of new diagnoses among PLWH in the 5 most recent years}_{j}$$

$$\boldsymbol{Additional lifetime HIV care and treatment costs (1 year)}_{\boldsymbol{j}}={Total number of additional cases (1 year)}_{j}\times Per person lifetime HIV care and treatemtent costs$$

$$\boldsymbol{Additional lifetime HIV care and treatment costs (5 years)}_{\boldsymbol{j}}={Total number of additional cases (5 year)}_{j}\times Per person lifetime HIV care and treatemtent costs$$
